# Novel *in vitro* and *in vivo* anti-*Helicobacter pylori* effects of pomegranate peel ethanol extract

**DOI:** 10.14202/vetworld.2021.120-128

**Published:** 2021-01-16

**Authors:** Amal Mayyas, Mohammad Abu-Sini, Rula Amr, Rand T. Akasheh, Waleed Zalloum, Ayman Khdair, Islam Hamad, Talal Aburjai, Rula M. Darwish, Luay Abu-Qatouseh

**Affiliations:** 1 Department of Pharmaceutical Sciences, Faculty of Pharmacy, University of Jordan, 11914 Amman, Jordan; 2 Department of Pharmacy, Faculty of Health Sciences, American University of Madaba, 11821 Madaba, Jordan; 3 Department of Pharmacy, Faculty of Pharmacy, Al-Zaytoonah University of Jordan, Amman, Jordan; 4 Department of Pharmacology and Biomedical Sciences, Faculty of Pharmacy, University of Petra, 961343 Amman, Jordan

**Keywords:** *Helicobacter pylori*, *in vitro*, *in vivo*, pomegranate peel ethanol extracts, urease inhibition.

## Abstract

**Background and Aim:**

Interest in plants with antimicrobial properties has been revived due to emerging problems associated with using antibiotics to eradicate *Helicobacter pylori*. Accordingly, this study aims to assess the antibacterial effects of *Punica granatum* and the possible synergistic effect of its extract along with metronidazole against *H. pylori*.

**Materials and Methods:**

Pomegranate peel ethanol extracts (PPEE) was tested against a control strain of *H. pylori* (NCTC 11916) *in vitro* and *in vivo* in female Wistar rats. Moreover, the synergistic effect of PPEE in combination with metronidazole was tested *in vitro*.

**Results:**

The PPEE exhibited a remarkable activity against *H. pylori* with a minimum inhibitory concentration (MIC) of 0.156 mg/mL. Furthermore, the extract exhibited a pronounced urease inhibitory activity (IC_50_ ~6 mg/mL) against the tested strain. A synergistic effect between PPEE and metronidazole was also observed (fractional inhibitory concentrations <0.5). Oral treatment of rats with PPEE for 8 days produced a significant reduction in *H. pylori* gastritis and a significant decrease in both lymphocytic and positive chronicity.

**Conclusion:**

Pomegranate extract is probably safe and represents a potential alternative and complementary therapy for reducing *H. pylori* associated with gastric ulcers.

## Introduction

Peptic ulcer disease caused by *Helicobacter pylori* infection is a worldwide problem [1]. *H*. *pylori* can weaken the protective mucus coating of the stomach and the upper part of the small intestine, making them more vulnerable to the acidic gastric juices. This could lead to gastritis and ulcers. Other complications include chronic gastritis, gastric carcinoma, and primary gastric B-cell lymphoma [2].

Ancient civilizations used medicinal plants to treat a wide range of diseases. And in modern medicine, these plants represent a rich source for drug discovery [3]. Plant extracts contain bioactive compounds that can be used prophylactically and therapeutically over synthetic drugs [4,5] and may display potent antimicrobial properties [6]. *Punica granatum* or Pomegranate is one plant with great therapeutic potential. Pomegranate peel extract with its high antioxidant capacity exhibits anti-inflammatory and anti-ulcerogenic activities that are ten-fold higher than those of the pulp extract [7]. Moreover, research on the synergetic activity of pomegranate peel extract is sparse.

Accordingly, and as part of the Jordan traditional medicine project, the present study was designed to evaluate the anti-*H. pylori* and urease inhibitory activities of pomegranate peel ethanol extracts (PPEE) extract, and to investigate possible synergism between PPEE and metronidazole against this bacteria.

## Materials and Methods

### Ethical approval

This study was approved by the animal care committee at the University of Jordan.

### Study period and location

This research was conducted for 8 months (between October 2015 and July 2016) at University of Jordan, Jordan.

### Chemicals and laboratory consumables

Chemicals and solvents used in all experiments were of analytical grade. Solvents, including ethanol (EOH) and Dimethyl sulfoxide (DMSO), were purchased from LABCHEM (USA) and Fisher chemical (UK), respectively. Sterile blank disks of 6-mm diameter and disks with standard antibiotics (clarithromycin 15 μg, metronidazole 5 μg, ciprofloxacin 5μ g, and tetracycline 30 μg) were purchased from Oxoid (UK).

### Plant extraction

Peels of *P. granatum* (Pomegranate) were collected from North Jordan and identified by direct comparison with the authenticated sample at the herbarium of the Faculty of Science and the Faculty of Agriculture at The University of Jordan. A voucher specimen was deposited with a voucher number of Pun-001.

Peels were air-dried under ambient conditions for 7 days, then powdered using an electric grinder and stored in plastic bags. Approximately 500 g of finely-powdered peels were soaked in 70% EtOH in a closed container at room temperature at a ratio of 1:10 for 3 days. The pooled solvent was evaporated and produced a thick viscous pellet, which was further oven-dried at 50°C overnight, making a 27.77 g% yield w/w.

### *H. pylori* isolation, culture media, and growth conditions

Twelve *H. pylori* clinical isolates and one reference strain of *H. pylori* (NCTC 11916) were used in this study. The clinical strains were isolated from gastric biopsy samples obtained by a gastroenterologist at the Jordan University Hospital during a routine endoscopy. Gastric biopsies were processed according to standard methodology [8]. Briefly, each biopsy was homogenized using a tissue homogenizer (IKA, Staufen, Germany). Aliquots of 100 μL of the homogenate were primarily cultured on Columbia blood agar (Oxoid, Hampshire, UK) supplemented with 7% (v/v) horse blood and Dent selective supplement (Oxoid, Hampshire, UK). The same bacterial plates were used for subcultures but without the Dent supplement. All plates were incubated at 37 °C under microaerophilic conditions using the CampyGen atmosphere generating system (Oxoid, Hampshire, UK) in anaerobic jars for 5-7 days. The growth of *H. pylori* was confirmed according to biochemical tests (positive for oxidase, catalase, and urease). *H. pylori* cultures were stored at −70°C in trypticase soy broth (Oxoid, Hampshire, UK) containing 10% (v/v) fetal calf serum (PAA, Pasching, Austria) and 15% glycerol [1,8]. All experiments were performed in triplicates to ensure consistency of the results.

### Determination of *H. pylori* inhibition zones

The disk diffusion method was used to study the susceptibility of *H. pylori* to PPEE. The *H. pylori* suspension was prepared and adjusted to 6×10^8^ CFU/mL (2 McFarland) in a fixed volume of 100 μL as described by Tayseer *et al.* [1] and Abu-Sini *et al*. [8]. The bacterial suspension was cultured on solid Columbia blood agar media. The PPEE stock solution was prepared by dissolving 2.5 g of the dry extract in a 2:1 (v/v) DMSO-phosphate-buffered solution (PBS) (pH 6.8) to obtain 2.5 w/v %. Thereafter, 30 μL of this extract were used to impregnate each of the sterile blank disks. These disks were left to dry, then placed on the *H. pylori* culture plates and incubated at 37°C for 1 h [9]. Disks with standard antibiotics (clarithromycin 15 μg, metronidazole 5 μg, ciprofloxacin 5 μg, and tetracycline 30 μg) were used as positive controls whereas, disks impregnated in a 2:1 (v/v) DMSO-PBS (pH 6.8) only were used as negative controls [1]. Finally, zones of inhibition were measured and recorded.

### Determination of minimum inhibitory concentration (MIC) of PPEE

The two-fold agar dilution method was used to determine the MIC of PPEE against *H. pylori*. This was conducted according to the guidelines established by the Clinical and Laboratory Standards Institute [10]. The PPEE stock solutions were diluted in PBS (pH 6.8) by the following order: 1:2, 1:4, 1:8, 1:16, 1:32, 1:64, 1:128, 1:256, 1:512 1: 1024, 1:2048, 1:4096, and 1:8192. Then, 1 mL of each diluted fraction was incorporated into 19 mL molten Columbia blood agar plates to final concentrations of 0.00061-1.25 mg/mL. Three μL of *H. pylori* (6×10^8^ CFU) were applied on the surface of each plate and allowed to be dried before incubation.

### *In vitro* synergism effect between PPEE and metronidazole against *H. pylori*

Checkerboard test was used to investigate the synergism between PPEE and metronidazole [1,8]. PPEE (0.1 g/mL) was dissolved in 2:1 (v/v) DMSO-PBS (pH 6.8) then serially diluted to obtain 25, 12.5, 6.25, 3.125, 1.563, 0.7813, 0.3906, 0.1953, and 0.0977 mg/mL. Furthermore, 51.2 mg of metronidazole was dissolved in 10 mL of 1:1 (v/v) DMSO-distilled water followed by serial dilutions in PBS (pH 6.8) to obtain 5.12, 2.56, 1.28, 0.64, 0.32, 0.16, 0.08, and 0.04 mg/mL. Thereafter, 1000 or 500 μL from each diluted preparation was taken and mixed with 9.5 mL soft Columbia blood agar media. Then, fractional inhibitory concentrations (FICs) were calculated using the equation bellow. The FIC indices were interpreted as synergy if <0.5; additive if .0.5-1.0; indifference if 1.0-4.0; and antagonism if >4.0 [8].

FIC = (MIC of drug A in combination/MIC of drug A alone) + (MIC of drug B in combination/MIC of drug B alone).

### Quantitative determination of urease inhibition concentration (IC_50_)

Urease inhibition assay was performed as described by Nagata *et al*. [11], where urease inhibition served as a proxy for *H. pylori* inhibition. A 10 μL of 6×10^8^ CFU/mL *H. pylori* suspension was challenged with 200 μL of a detection reagent composed of 50 mM PBS (pH 6.8) containing 500 mM urea and 0.02% phenol red. Color development was monitored by measuring the O.D. at 555 nm wavelength in a 5-min interval. Samples of bacteria with a detection reagent only or PPEE with a detection reagent only served as controls. The following equation calculates the percentage of inhibition:

% inhibition = ([activity without plant extract - activity with plant extract]/[activity without plant extract])×100.

A lack of potential urease inhibition was indicated if IC_50_ was more than 12.5 mg/mL (inhibition of <60%). The IC_50_ of PPEE was determined using ED_50_ plus (v1.0) software (Vergas, 2000). The urease inhibitory activity of PPEE was compared to the reference urease inhibitor acetohydroxamic acid (AHA) from Sigma (USA).

### Induction of *H. pylori* infection in vivo

Female Wistar rats were selected according to Crabtree *et al*. [12] selection criteria. Wistar rats were housed in the animal house of the University of Jordan, fed a commercial diet (Hammodeh factory, Jordan) *ad libitum* and given sterile tap water. A total of 45 8-week-old female Wistar-rats (197.7±1.38 g) were pre-treated with streptomycin suspended in tap water (5 mg/mL) for 3 days before the first *H. pylori* inoculation. After 1 day of fasting, rats were inoculated by gavage 1 mL/rat with *H. pylori* suspension of 9 McFarland (2.7×10^9^ CFU/mL) twice daily at an interval of 4 h [13], and for 8 consecutive days. Control rats were inoculated with sterile PBS (non-infected group). Eight weeks after the last inoculation, rats were sacrificed, and their stomachs were removed and sliced into three parts. The antrum region was used for the urease test, the second part for bacterial colonization and inflammatory response by culture, and rapid urease test. Another part of the stomach was rinsed in PBS and homogenized using a tissue homogenizer (Germany). Thereafter, aliquots of 100 mL of the homogenate were cultured, as described above, following similar growth conditions. The remaining stomach part was fixed in 10% formalin. Samples were sent out to Alpha Medical for hematoxylin and eosin (H&E) staining and histopathological analysis. Updated Sydney System with visual scales was used as a reference standard grading system. The density of *H. pylori* indicated if the case is acute infection, chronic inflammation, intestinal metaplasia, or atrophy [3,14].

Infection with *H. pylori* was indicated if the rat met two or three of the following criteria: (a) A positive urease test of gastric biopsy; (b) a positive *H. pylori* growth in the cultured gastric biopsy; and/or (c) the presence of *H. pylori* curved bacilli in the gastric tissue by standard histological examination [13].

### Acute toxicity test of pomegranate extract

Acute toxicity was evaluated *in vivo* in female Wistar rats. Tests were performed initially to ascertain the safety of the prepared doses of PPEE. According to the guidelines of the Organization for Economic and Cooperation and Development for testing of chemicals [15], three different doses of 50, 300, and 2000 mg/kg were administered orally through gastric gavage to three different groups of rats (n=3 per group) for the determination of the lethal dose that kills 50% of the test animals in a group (LD_50_).

Rats were weighed before, during, and after the procedure to measure any changes in body weights. Rats were also monitored to observe any possible changes in their behaviors after dosing at least once during the first 30 min and periodically through 24 h. Special attention was given during the early 4 h and daily for 14 days. Rats were monitored to observe any signs of toxicity including changes in skin and eyes, breathing issue, seizures, or death.

### Testing the effect of PPEE on *H. pylori*

Treatment with PPEE was initiated after 7 weeks of the last *H. pylori* inoculation. The treatment was prepared by dissolving 1250 mg of PPEE in 100 mL water. A PPEE dose 50 mg/kg was administered twice daily for 8 days. The treatment was chosen based on a published protocol by Moghaddam *et al*. [7]. The PPEE dose represents 1/40 of the highest safe dose tested in the acute toxicity study (2000 mg/kg).

Rats were randomly assigned into the following groups: (1) Non-infected treated (n=9); (2) infected non-treated (n=13); (3) infected treated (n=13); and (4) non-infected non-treated (n=10). Imbalance in the number of rats per group is attributed to their housing distribution into cages.

### Statistical analysis

Chi-square test was used to test the difference between rat groups. p<0.05 was set to indicate statistical significance. Microsoft Windows 7 Excel program was used to conduct the analysis.

## Results

### Pomegranate peel extract exhibits anti-*H. pylori* activity *in vitro*

The antibacterial activity of PPEE against the control *H. pylori* strain of as represented by the inhibition zone was 32.53±0.33 mm, compared to 65 mm, 56 mm, and 14 mm of ciprofloxacin, tetracycline, and clarithromycin, respectively, while no *H. pylori* inhibition by metronidazole was observed (Table-1).

**Table-1:** Inhibition zones (mm) of PPEE (0.75 mg/disc) against clinical strains of *H. pylori* (1–12) and the control strain.

Experiment number	Zones of inhibition (mm) of clinical isolates												Control Strain (NCTC 11916)

	1	2	3	4	5	6	7	8	9	10	11	12	
Trial 1	20	19.8	32.3	29.2	28.2	24.8	29.2	30.2	23.4	29.5	28.6	31.1	33
Trial 2	18.8	19.3	30.5	30.6	28.3	26.1	27.8	28.2	22.8	29.2	27.8	30.2	32.7
Trial 3	19.1	22	31	32.1	30.1	25.4	29	32	22.7	30	30.1	31	31.9
Average	19.3	20.37	31.27	30.63	28.87	25.43	28.67	30.13	22.97	29.57	28.83	30.77	32.53
±SEM	±0.36	±0.83	±0.54	±0.84	±0.62	±0.38	±0.44	±1.09	±0.22	±0.23	±0.67	±0.28	±0.33
CIP	20	60	0	50	53	65	59	0	0	64	50	55	65
MTZ	0	0	0	0	0	0	0	0	0	0	0	0	0
DMSO-PBS	0	0	0	0	0	0	0	0	0	0	0	0	0

PPEE=Pomegranate peel ethanol extracts, CIP=Ciprofloxacin, MTZ=Metronidazole, DMSO=Dimethyl sulfoxide, PBS=Phosphate buffer solution

The average MIC values of PPEE against the clinical *H. pylori* strains were 0.163±0.04 mg/mL. In comparison, The MICs of ciprofloxacin and metronidazole (positive controls) were 0.00043±6.0×10^-5^ and 0.442±0.035 mg/mL, respectively (Table-2). Furthermore, the MIC of PPEE against the control *H. pylori* strain was 0.156 mg/mL as compared to 0.0006 mg/mL for ciprofloxacin, while the strain was resistant to metronidazole with a MIC of 0.256 mg/mL. The IC_50_ for urease inhibition by PPEE was 6.3 mg/mL.

**Table-2:** Minimum inhibitory concentration of PPEE against clinical strains of Helicobacter pylori (1-12) and the control strain.

Experiment number	Minimum inhibitory concentration (MIC, mg/mL)												Control strain (NCTC 11916)

	1	2	3	4	5	6	7	8	9	10	11	12	
Trial 1	0.3125	0.625	0.0781	0.3125	0.0781	0.156	0.0781	0.0781	0.0781	0.156	0.0781	0.0781	0.156
Trial 2	0.3125	0.625	0.0781	0.156	0.0781	0.156	0.0781	0.156	0.0781	0.156	0.0781	0.156	0.156
Trial 3	0.625	0.3125	0.156	0.156	0.156	0.0781	0.156	0.0781	0.3125	0.0781	0.156	0.0781	0.156
Averge	0.416	0.521	0.104	0.208	0.104	0.130	0.104	0.104	0.156	0.130	0.104	0.104	0.156
± SEM	±0.104	±0.104	±0.026	±0.052	±0.026	±0.026	±0.026	±0.026	±0.078	±0.026	±0.026	±0.026	
Clinical strains													
Average minimum inhibitory concentration±SEM=0.163±0.04													
CIP	0.0006	0.0006	0.0003	0.0006	0.0003	0.0004	0.0006	0.0004	0.00008	0.00004	0.0006	0.0006	0.0006
MTZ	0.256	0.512	0.512	0.512	0.512	0.512	0.256	0.256	0.512	0.512	0.512	0.512	0.256

PPEE=Pomegranate peel ethanol extracts, CIP=Ciprofloxacin, MTZ=Metronidazole, DMSO=Dimethyl sulfoxide

### Pomegranate peel extract and metronidazole exhibit synergistic activity against *H. pylori*

According to the standard checkerboard test, the MIC for PPEE was 0.156 mg/mL, and the MIC for PPEE in combination with metronidazole was 0.039 mg/mL. The MIC for metronidazole was 0.256 mg/mL, and the MIC for metronidazole in combination with PPEE was 0.016 mg/mL. The FIC values and FIC index showed a synergic effect for the combination (<0.5), where metronidazole FIC value mean (0.0625), PPEE FIC value mean (0.25), and FIC index (0.313). This indicates that the combination of PPEE with metronidazole was synergic.

Besides, PPEE was fractionated by column chromatography using solvents differing in their polarity; hexane, chloroform, ethyl acetate, butanol, and water. Fractions were assessed for their potential anti*-H. pylori* activity using antimicrobial susceptibility tests and *in vitro* interaction between the isolated compounds and Metronidazole (Synergism). A total of 0.3 mg/disk of butanol fraction displayed the highest inhibition zone of 15.6±0.3 mm among other fractions. Moreover, we performed a phytochemical analysis of the butanol fraction, which revealed the presence of gallic acid, mannitol sugar, and 5-hydroxy methyl furfural. Gallic acid, the major component in this fraction showed weak anti-*H. pylori* activity. On the other hand, gallic acid did not show any synergistic activity with metronidazole. Furthermore, 5-hydroxy methyl furfural was not tested against *H. pylori* as it was present in very minute amount in the butanol extract.

### Acute in vivo administration of pomegranate peel extract shows no signs of toxicity

During the 14 days of the experiment, even with a high PPEE dose of 2000 mg/kg, no significant change in rats’ weight compared with the control group was noticed (p>0.05). Moreover, no signs of toxicity or deaths were reported. The LD_50_ value was indeterminable even at the highest PPEE dose, which indicates that PPEE is non-toxic at doses of expected efficacy. Results show that the extract is non-toxic even at relatively high concentrations (Table-3). Based on these findings, the starting PPEE dose was selected at 1/40^th^ of 2000 mg/kg or 50 mg/kg.

**Table-3:** Signs of acute *in vivo* toxicity in response to PPEE.

Group	n	PPEE dose (mg/kg)	Average weight before treatment ±SEM (g)	Average weight after treatment ±SEM (g)	Number of dead rats
1	3	2000	260±16.3	262.5±21.6	0
2	3	300	263.75±20.6	280±11.5	0
3	3	50	250±26.3	253.75±11.1	0

PPEE=Pomegranate peel ethanol extracts

### Pomegranate peel extract inhibits *H. pylori* infection and pathology in vivo

#### Infected non-treated group

**Figure-1:**
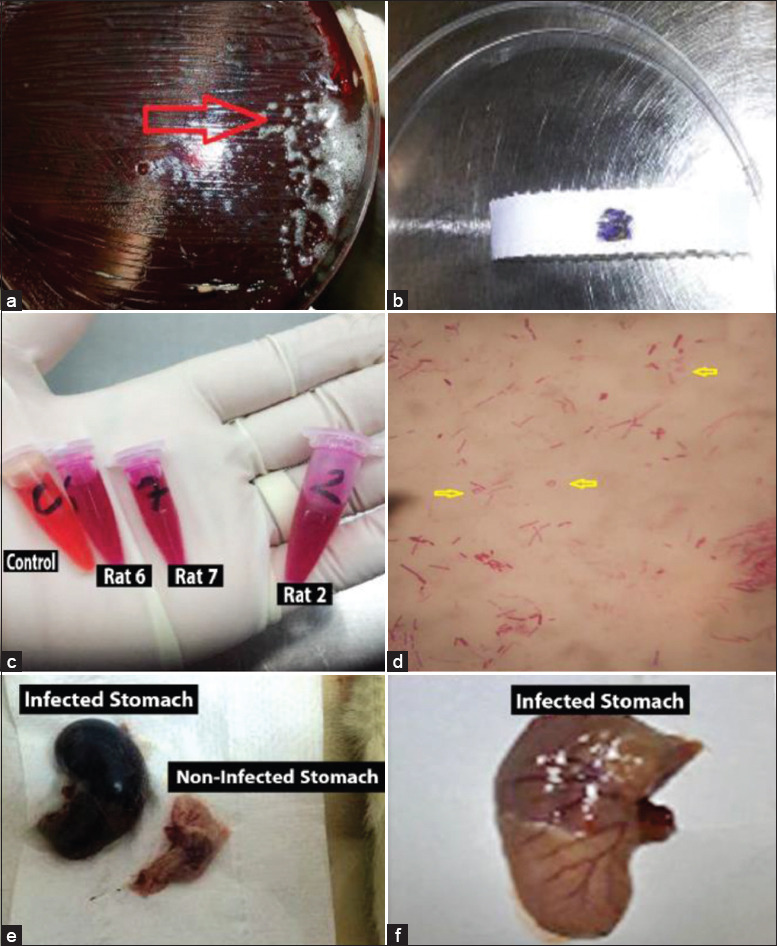
Indications of *Helicobacter pylori* infection in rat stomach. *H. pylori* positivity in cultures of gastric biopsies of rats as indicated by (a) catalase; (b) oxidase; (c) and urease activities; and (d) Gram (–ve) staining of selected colonies, where *H. pylori* looks spiral, as curved rods, coccoidal or U-shaped. (e) and (f) Stomachs from infected versus non-infected rats.

**Figure-2:**
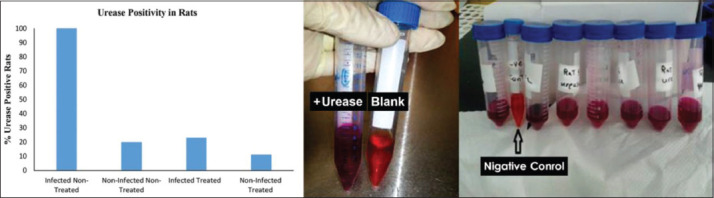
Efficacy of pomegranate peel extract in inhibiting urease activity *in vivo*. Left: Percentage of rats with urease activity in the antral stomachs in four experimental groups. Data are expressed as mean±SEM. p<0.05 between infected non-treated and infected treated groups. Right: Positive urease activity detected in the antral stomach of infected non-treated rats.

**Figure-3:**
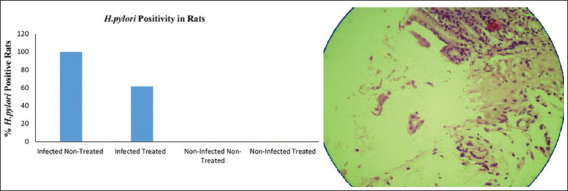
Efficacy of pomegranate peel extract in eradicating *Helicobacter pylori* *in vivo*. Left: Percentage of *H. pylori*-positive rats in four experimental groups. Data are expressed as mean±SEM. p<0.05 between infected non-treated and infected treated groups. Right: H&E-stained section of rat stomach showing *H. pylori* Gram (−ve) curved rods.

**Figure-4:**
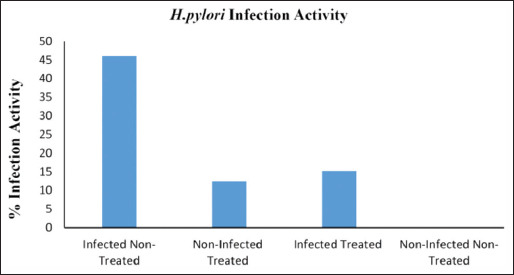
The effect of pomegranate peel extract on *Helicobacter pylori* infection chronicity *in vivo*. Left: Percentage of rats with *H. pylori* Chronicity in H&E-stained stomach sections in four experimental groups. Data are expressed as mean±SEM. p<0.05 between infected non-treated and infected treated groups. Right: Chronic gastritis in a section of H&E-stained stomach from an *H. pylori*-infected rat, with typical mixed lymphoid reaction in the gastric mucosa.

**Figure-5:**
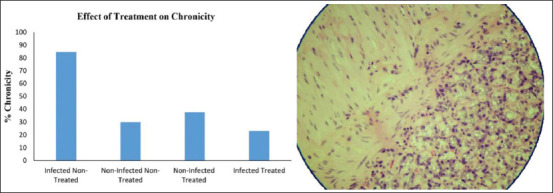
The effect of pomegranate peel extract on *Helicobacter pylori* infection activity *in vivo*. Percentage of rats with active infections as evaluated on H&E-stained sections of stomachs from four experimental groups. Data are expressed as mean±SEM. p<0.05 between infected non-treated and infected treated groups.

Gastric culture from all infected rats showed well-identified *H. pylori* colonies, positive Gram stain microscopic test, and positive catalase, and oxidase and urease activity tests. Specimens showed contamination with *Candida*, and other bacteria and spores and dysplasia were noticed in some infected stomachs (Figure-1). Bacterial urease activity was detected for each antral stomach part by developing an intense pink color for the urease reagent within 24 h (Figure-2). Histopathological analysis revealed *H. pylori* positivity in 100% of stomach specimens. Active infection was also found in 46.1% of the samples and positive chronicity in 84.6% (Figures-3-5, Table-4).

**Table-4:** Histopathological and urease tests of rat stomachs after 8 weeks of the last *H. pylori* inoculation.

Rat groups	Number of *Helicobacter pylori* +ve rats/n				Number of urease +ve Rats/n (%)
Infected non-treated	13/13				13/13 (100%)
	Infection activity 6/13	chronicity 11/13	Lymphocyte 5/13	neutrophils 11/13	
Infected treated	8/13				3/13 (23%)
	Infection activity 2/13	Chronicity 3/13	Lymphocyte 10/13	neutrophils 12/13	
Non-infected non-treated	0/10				2/10 (20%)
	Infection activity 0/10	chronicity 3/10	Lymphocyte 5/10	neutrophils 7/10	
Non-infected treated	0/9				1/9 (11.1%)
	Infection activity 1/9	Chronicity 3/9	Lymphocyte 7/9	Neutrophils 9/9	
	Infection activity 9/45	chronicity 20/45	Lymphocyte 27/45	Neutrophils 39/45	

#### Infected treated group

Urease positivity was observed in 23% of the gastric samples. Moreover, 61.5% of histopathological specimens showed *H. pylori* positive gastric mucosa. Chronicity and infection activity were observed in 23% and 15.3% of rat specimens, respectively (Table-4).

Chi-square test indicated a significant difference (p<0.05) between infected treated and non-infected treated rats in terms of urease activity (Figure-2), treatment effects on eradicating *H. pylori* (Figure-3), the chronicity of gastric infection, and the percentage of infection activity (Figures-4 and 5). This indicates that PPEE treatment displayed potent anti-*H. pylori* activity.

#### Non-infected treated group

Gastric culture showed no characteristic *H. pylori* Colonies in all samples. We observed *Candida* and other bacterial contamination. Moreover, 88.9% of gastric samples were urease negative, and histopathology results showed gastric mucosa with no *H. pylori* in 100% specimens. Chronicity and infection activity were found in 33.3% and 11.1% of the samples, respectively (Table-4).

#### Non-infected non-treated group

The gastric culture showed no characteristic *H. pylori* colonies in all samples. We observed contamination of *Candida* and other bacteria. While 80% of gastric samples were urease negative, all histopathological sections of gastric mucosa were *H. pylori* negative. Chronicity was detected in 30% of these samples (Table-4). Specimens showed *Candida*, other bacteria, and spores likely due to contamination.

## Discussion

We demonstrated that pomegranate peel extract displays a significant inhibitory effect on *H. pylori* growth and urease enzyme activity. To the best of our knowledge, this is the first report on the potential inhibitory properties of PPEE against urease enzyme activity, highlighting a possible mechanism through which pomegranate peel extract inhibits *H. pylori*. Similar findings were reported using pomegranate peel methanol extract with an average inhibition zone of 39 mm at 0.1 mg/disk using the disc diffusion method [16]. Furthermore; higher MIC values of 0.625-0.780 mg/mL were reported [17]. The differences between our findings and the values reported in the literature can be attributed to the type of extract and the variability in the *H. pylori* strains used and their susceptibility or resistance to antimicrobial agents [18].

Combining PPEE with metronidazole reduced the MIC for this antibiotic by four-folds. This suggests that effective therapy may be achieved with this antibacterial combination. Interestingly, a study by Voravuthikunchai *et al*. [17] reported that Pomegranate fruit rind extract displays anti-adhesive effects against *H. pylori* to gastric mucosa by altering the bacterium’s cell surface hydrophobicity, which may synergistically facilitate the elimination of bacterial cells from the human body. However, in our study, we did not investigate the mechanisms behind the synergistic activity between metronidazole and PPEE. Future studies must focus on this aspect.

The model used by Werawatganon emphasized the importance of CFU per dose, the number of inoculations, and the pretreatment with antibiotics. We used this model of *H. pylori* infection to mimic human mild chronic gastritis and study the efficacy of PPEE against *H. pylori* infection [13]. In our study, *H. pylori* colonization was successfully established in all *H. pylori* inoculated-rats with an infection rate of 100%. This was statistically significant compared to non-infected controls (p<0.05). Compared to the previous works on Wistar rats, Li *et al*. [19] reported successful colonization of *H. pylori* in rats and mild-to-moderate mucosal inflammation. In a rat model of chronic *H. pylori* infection, Werawatganon reported a 69.8-83.0% success rate of infection [13]. However, our model achieved a higher colonization rate than what was reported in the literature. A possible explanation of this could be attributed to the repeated inoculation of *H. pylori* during the protocol. Our data demonstrate that the protocol we adopted is not only reliable for establishing *H. pylori* infection in Wistar rats but also for conducting *in vivo* experiments for *H. pylori* eradication by herbal extracts. Thus, our modified rat model can be used to assess potential therapies for treating human *H. pylori* infection and to study *H. pylori*-associated gastrointestinal pathology.

Based on the *in vitro* *H. pylori* and urease inhibition activities of PPEE, we used this extract to treat H*. pylori*-infected Wistar rats. To the best of our knowledge, this is the first report to show that PPEE can treat *H. pylori* infection *in vivo*. Other studies reported the potential usefulness of Pomegranate fruit juice or rind (REF). Moghaddam *et al*. [7] reported that the hydroalcoholic extract of Pomegranate fruit peel exhibits potential antiulcer properties in Wistar rats. Such efficacy was attributed to the high antioxidant activity of Pomegranate fruit extract [7]. Future studies may focus on exploring the bioactive ingredients in pomegranate peel that may be responsible for its anti-*H. pylori* effects. Matsubara *et al*. [20] found that the antioxidative flavonoid epigallocatechin gallate (the main component in green tea extracts) exhibits potent *H. pylori* urease inhibitory activity with an IC_50_ value of lower than that of acetohydroxamic acid *in vitro*. Notably, the most abundant catechins in Pomegranate are the potent antioxidant epicatechin and epigallocatechin gallate [21]. This hints that the anti-*H. pylori* effects of pomegranate peel may at least in part be attributed to its catechins.

In our study, PPEE treatment led to a modest reduction in *H. pylori* colonization and infection in Wistar rats (~39% treatment rate, p<0.05) after 8 days of treatment with a PPEE dose of 50 mg/kg. The treatment also significantly reduced infection chronicity (p<0.05). Furthermore, the anti-inflammatory effect of PPEE on gastric mucosa was remarkable. Since PPEE demonstrated no signs of acute toxicity at much higher doses (2000 mg/kg), it would be interesting to test the potency of higher PPEE doses (>50 mg/kg) against *H. pylori*. This study is superior to other studies that used Pomegranate extracts for anti-ulcerogenic and anti-inflammatory activities in non-*H. pylori* models [6,7].

## Conclusion

Pomegranate peel extract exhibited remarkable activity against *H. pylori* *in vitro* with a MIC of 0.156 mg/mL, and a pronounced urease inhibitory activity with an IC_50_ of ~6 mg/mL against the tested strain. We found a synergic effect of PPEE-metronidazole combination against *H. pylori* with an FIC of <0.5. Oral treatment of rats with PPEE for 8 days led to a significant reduction in *H. pylori* gastritis, in addition to a significant decrease in both lymphocytic and positive chronicity. Importantly, PPEE showed no signs of acute *in vivo* toxicity even at relatively high doses. Therefore, we propose PPEE as a potential alternative or complementary therapy to treat *H. pylori* infection associated with clinical symptoms, but more investigations are needed.

## Authors’ Contributions

AM and MAS performed the experiments. AM wrote the paper. LA, RMD, and AK conceived and designed the microbiological and biological activities. TA and WZ designed the extracted solutions and their corresponding experiments. RA, RTA, and IH analyzed and interpreted the data. All authors have read and approved the final manuscript.
